# Child Welfare in Birmingham

**Published:** 1920-02-14

**Authors:** 


### Child Welfare in Birmingham.

BIRMINGHAM, which always does things in the grand manner, has prepared a comprehensive scheme for extending the maternity and child-welfare work. Meanwhile the Corporation is dealing with the question of the supply of milk to nursing and expectant mothers and children under five. Its scheme is to supply at the following reduced prices: 4d. a quart where the family income, after deducting rent, amounts to less than 7s. 6d. per head per week, and 8d. per quart where the income is over 7s. 6d. but does not exceed 10s. It is not possible to indicate the cost, but the Corporation proposes to set aside £10,000 in its yearly estimates for this purpose.

